# Metabolic profiling reveals altered sugar and secondary metabolism in response to *UGPase* overexpression in *Populus*

**DOI:** 10.1186/s12870-014-0265-8

**Published:** 2014-10-07

**Authors:** Raja S Payyavula, Timothy J Tschaplinski, Sara S Jawdy, Robert W Sykes, Gerald A Tuskan, Udaya C Kalluri

**Affiliations:** BioEnergy Science Center and Biosciences Division, Oak Ridge National Laboratory, Oak Ridge, TN 37831 USA; The Biosciences Center, National Renewable Energy Laboratory, Golden, CO 80401 USA

**Keywords:** Metabolic profiling, Primary and secondary metabolism, Cell wall, UGPase, *Populus*

## Abstract

**Background:**

UDP-glucose pyrophosphorylase (UGPase) is a sugar-metabolizing enzyme (E.C. 2.7.7.9) that catalyzes a reversible reaction of UDP-glucose and pyrophosphate from glucose-1-phosphate and UTP. UDP-glucose is a key intermediate sugar that is channeled to multiple metabolic pathways. The functional role of *UGPase* in perennial woody plants is poorly understood.

**Results:**

We characterized the functional role of a *UGPase* gene in *Populus deltoides, PdUGPase2*. Overexpression of the native gene resulted in increased leaf area and leaf-to-shoot biomass ratio but decreased shoot and root growth. Metabolomic analyses showed that manipulation of *PdUGPase2* results in perturbations in primary, as well as secondary metabolism, resulting in reduced sugar and starch levels and increased phenolics, such as caffeoyl and feruloyl conjugates. While cellulose and lignin levels in the cell walls were not significantly altered, the syringyl-to-guaiacyl ratio was significantly reduced.

**Conclusions:**

These results demonstrate that *PdUGPase2* plays a key role in the tightly coupled primary and secondary metabolic pathways and perturbation in its function results in pronounced effects on growth and metabolism beyond cell wall biosynthesis of *Populus*.

**Electronic supplementary material:**

The online version of this article (doi:10.1186/s12870-014-0265-8) contains supplementary material, which is available to authorized users.

## Background

Sucrose is the primary product of photosynthesis and the initial form of transported sugar in most plants. In higher plants, sucrose is hydrolyzed by either invertases to form glucose and fructose or sucrose synthases (SuSy) to form *uridine diphosphate glucose* (UDP-glucose) and fructose [1]. The carbon in glucose and fructose is further channeled into various primary or secondary metabolic pathways based on the spatiotemporal activity of metabolizing enzymes [2-4]. Sugar metabolizing enzymes have, therefore, been recognized as key gatekeepers of carbon allocation and partitioning pathways in plants [2,3]. The sugar, UDP-glucose, represents an important branch point in carbohydrate metabolism that can potentially be channeled directly for synthesis of starch, sucrose, cellulose or hemicellulose and pectin via synthesis of other nucleotide sugars such as UDP-*glucuronic* acid [2,3].

UDP-glucose pyrophopharylase (UGPase), also referred to as UGlcPP or *uridine triphosphate* glucose-1-P uridylyltransferase, is a sugar-metabolizing enzyme (E.C. 2.7.7.9) that catalyzes the reversible formation of UDP-glucose and pyrophosphate from glucose-1-phosphate and *uridine triphosphate* (UTP). In photosynthetically active mature leaves, UGPase catalyzes the reaction in the direction of UDP-glucose and sucrose via coupled action of sucrose phosphate synthase (SPS) [reviewed in 5]. However, in sink organs, which depend on imported sucrose, UGPase is proposed to function in the conversion of UDP-glucose, which is produced via SuSy hydrolysis of sucrose, to glucose-1-phosphate [5].

Based on an analysis of the most recent version of the *Populus trichocarpa* genome and previous reports from hybrid aspen (*Populus tremula x tremuloides*), rice (*Oryza sativa*) and *Arabidopsis thaliana*, *UGPase* genes appear to belong to small two-gene containing families [6-8].

UGPases have been purified from and expressed in several eukaryotic and prokaryotic organisms [9-11]. In vitro enzyme assays using AtUGPase1 and AtUGPase2 expressed in *Escherichia coli* suggested that both the isoforms have slight preference for the pyrophosphorylation direction of the reaction [12]. AtUGPase2 exhibited higher activity than that of AtUGPase1 in both directions, pyrophosphorolysis and synthesis [12]. The two isoforms showed no activity with other sugars such as ADP-glucose/ATP or galactose-1-P [12]. Antisense suppression of *UGPase1* in *Arabidopsis* was reported to have no effect on plant growth, however, soluble carbohydrate and starch content was found to be reduced [13]. Tobacco (*Nicotiana tabacum*) plants overexpressing *AxUGPase,* a *UGPase* gene from *Acetobacter xylinum*, exhibited increased plant height in several lines, similar total dry weight relative to controls and no significant changes in sugars, starch or cellulose levels [14]. However, overexpression of the same bacterial *UGPase* in hybrid poplar (*Populus alba* x *grandidentata*) resulted in significantly reduced growth, increased levels of soluble sugars, starch and cellulose, and reduced lignin [15]. Three studies involving antisense downregulation of a native *UGPase* gene in potato (*Solanum tuberosum*) led to contrasting and inconclusive results. An early report suggested that a strong 95% reduction in UGPase transcripts and enzyme activity had no apparent effects on plant growth and sugar levels [16]. In two subsequent studies, however, antisense suppression of *UGPase* in potato resulted in significantly lower sucrose levels [17,18]. Based on microarray analysis of cell types representing the discrete stages of xylem development and wood formation in *Populus*, native *UGPase* expression was enhanced during late xylem cell expansion and secondary cell wall formation, suggesting a potential functional role for UGPase in UDP-glucose substrate provision during secondary cell wall formation [19]. In total, these results imply distinct host-specific-functional activities of UGPase in alternate plant species. A functional genomics investigation of native *Populus UGPase* gene has not been reported.

Given that plant metabolism is tightly regulated for coordinated plant response to various internal and external cues, and carbon flow links primary metabolism to secondary metabolism, it is surprising how little we know about the enzymes interconnecting the primary and secondary metabolic pathways. It is plausible that manipulation of sugar metabolizing enzymes, such as UGPase, can affect both primary sugar and secondary wall carbohydrate metabolism; however, it is unclear if and how such manipulations may quantitatively and qualitatively affect secondary metabolic pathways of shikimate, phenylpropanoid and lignin biosynthesis. Therefore, we have undertaken a detailed metabolic characterization of *Populus deltoides* plants overexpressing a native *PdUGPase2* gene. Transgenic plants had pronounced growth and metabolic changes relative to control plants. The changes in cell wall properties were, however, subtle with significantly reduced syringyl-to-guaiacyl ratios as one of the few alterations in transgenic cell walls. Overall, our results support a role for PdUGPase2 in carbohydrate metabolism, secondary metabolism and plant growth and development.

## Results and discussion

### Phylogenetic analysis and expression of *UGPase* in *Populus*

**Figure 1:**
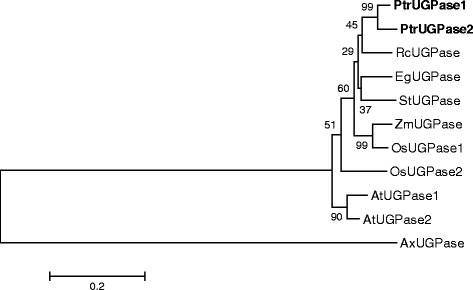
**Phylogenetic analyses of UGPases from** ***Populus*** **and other species.** Analysis was conducted in MEGA4 program using the Neighbor-Joining method. The percentage of replicate trees in which the associated taxa clustered together in the bootstrap test (500 replicates) are shown next to the branches. UGPases from this study are in bold. AtUGPase1 (*Arabidopsis thaliana*): AT3G03250; AtUGPase2: AT5G17310.2; AxUGPase (A*cetobacter xylinum*):AAA21888.1; EgUGPase (*Eucalyptus grandis*): ACF04278.1; OsUGPase1 (*Oryza sativa*): ABD57308; OsUGPase2: AF249880; PtrUGP1 (*Populus trichocarpa):* Potri.004G074400; PtrUGP2, Potri.017G144700; RcUGPase (*Ricinus communis*): XP_002526594.1; StUGPase (*Solanum tuberosum*): AAL99197.1; ZmUGPase (*Zea mays*): NP_001130742.1.

In several previous reports including *Populus*, the *UGPase* gene family has been reported to comprise of two genes [6-8]. A phylogenetic analysis was performed using two previously reported UGPase isoforms from *Populus* [8], orthologs from other plant species and AxUGPase as an outgroup. UGPase1 and UGPase2 cluster together closer than with any other UGPases (Figure 1). While PdUGPase1 and PdUGPase2, which appear to have arisen during the Salicoid duplication event [20], share more than 97% similarity (94% identity) with each other, they also share 92% similarity with AtUGPases vs. 90% with a gymnosperm UGPase (Additional file 1). There is a lower, 87%, identity between the rice paralogs, OsUGPase1 and OsUGPase2, and a 93% identity between the *Arabidopsis* paralogs, AtUGPase1 and AtUGPase2. The similarity of plant UGPases with the previously characterized AxUGPase averaged at 21% (Additional file 1). The nucleotide binding loop (NB-loop) and insertion loop (I-loop), involved in dimer formation and stabilization [21,22], are conserved in AtUGPase and PdUGPase but absent in AxUGPase (Additional file 2), suggesting a potentially distinct enzymatic mechanism in plants involving dimer- or-oligomerization of proteins. In support of this hypothesis, it has been shown that the activity of barley UGPase is regulated by di- or oligomerization status of the protein, with monomer status representative of the active form of UGPase enzyme [23,24].

**Figure 2:**
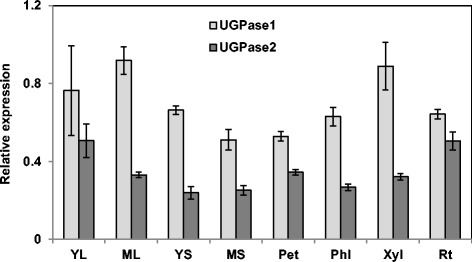
**Relative expression of** ***UGPase*** **isoforms in different tissues.** Expression of *UGPase1* and *UGPase2* in YL, young leaf, ML, mature leaf, YS, young stem, MS, mature stem, Pet, petiole, Phl, phloem, Xyl, xylem and Rt, root. Data represent means ± SE (n = 3). Relative expression was calculated based on the expression of reference genes *Ubiquitin-conjugating enzyme E2* (Potri.006G205700) and *18S ribosomal RNA* (AF206999)*.*

Expression of the two *PdUGPase* genes was quantified in eight *Populus* tissue types including young leaf (leaf plastochron index 1, LPI 1), mature leaf (LPI-6), young stem (internodes 1 to 3), mature stem (internodes 6 to 8), petiole of a mature leaf, phloem (bark), xylem (stem scrapings) and root by quantitative reverse transcriptase PCR (qRT-PCR). Both isoforms were expressed in all tissues, with an overall higher expression level of *UGPase1* relative to *UGPase2* (Figure 2). Among all the tissues profiled, expression of *PdUGPase1* was relatively higher in mature leaves and xylem, while expression of *PdUGPase2* was higher in young leaves and roots. Our results are consistent with previously published data involving alternate tissues and organs, which showed that *UGPase1* and *UGPase2* were abundant in flowers and young leaves [8]. In two additional studies, UGPase activity was also higher in xylem than in leaves [14,15]. In potato, where only one isoform has been reported, UGPase activity was abundant in sink tissues, such as stems and tubers [16]. In *Arabidopsis*, *UGPase1* was reported to have enhanced expression in leaves and stems compared to flowers, whereas *UGPase2* had enhanced expression in flowers [25].

### Morphological characterization of *PdUGPase2* overexpression plants

**Figure 3:**
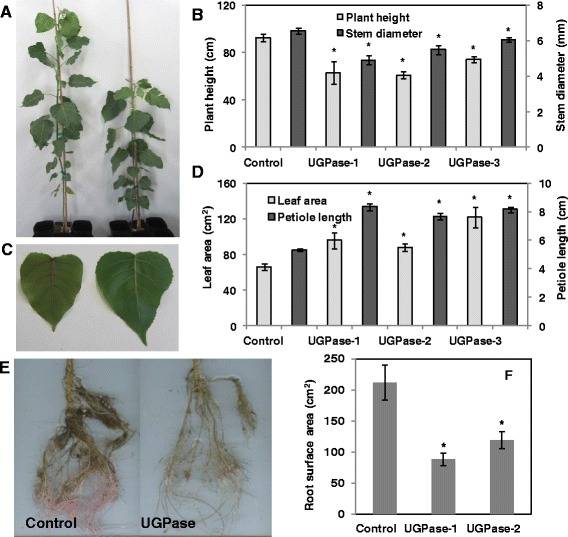
**Phenotypic characterization of** ***UGPase2*** **overexpression lines.** Differences in plant height **(A and B)** and stem diameter **(B)**, leaf area **(C and D)** petiole length **(D)** root system **(E)** and root surface area **(F)** among control and *UGPase2* transgenic plants. Data represent means ± SE (n ≥ 3). * indicates statistically significant, *p* ≤ 0.05 based on Student’s *t*-tests.

**Figure 4:**
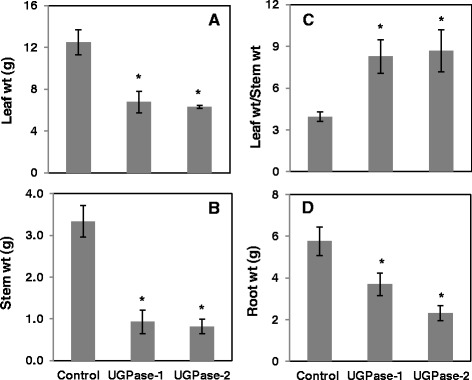
**Dry weight of different plant parts in control and two** ***UGPase2*** **transgenic lines.** Dry weights corresponding to leaf **(A)**, stem **(B)**, and root **(D)** and the ratio of leaf to stem dry weight **(C)** are presented. Data represent means ± SE (n = 3). *indicates statistically significant, *p* ≤ 0.05 based on Student’s *t*-tests.

Transgenic *Populus deltoides* plants overexpressing the *PdUGPase2* gene (Potri.017G144700) under the control of a constitutive Polyubiquitin 3 (AtUBQ3, Accession L05363) promoter were maintained and characterized under greenhouse conditions. In an initial transgenic phenotyping study, all *PdUGPase2* overexpression lines were found to be consistently shorter in height (20 to 50%) with thinner stems (37 to 47%) compared to control plants (Additional file 3). Assessment of an independently grown second set of transgenic plants confirmed that plant height and stem diameter were consistently smaller relative to controls (Figure 3A and B). Conversely, leaf area (35 to 55%) and petiole length (45 to 55%) were significantly greater in transgenics (Figure 3C and D), though total leaf dry weight was reduced as a result of fewer leaves (Figure 4A). Additionally, reduced plant height and stem diameter resulted in a significant reduction in stem dry weight (Figure 4B). When compared to controls, leaf-to-stem biomass ratio doubled (Figure 4C), whereas root weight and root surface area were reduced (50%) in transgenic plants (Figures 3E, F and 4D). We quantified *UGPase2* transcript levels in mature leaf petioles of three selected lines and found that *PdUGPase2* expression increased 2-fold (Additional file 4). Our results suggest that modest changes in native UGPase2 levels can have pronounced morphological effects on plant phenotype in woody perennials such as *Populus*.

Previous studies of suppression or overexpression of *UGPase* in other plant species have reported phenotypes ranging from no relative change to severely reduced plant height. In potato, for example, despite a strong 96% reduction in UGPase enzyme activity as a result of antisense suppression, plant growth or tuber development was found to be unaffected [16]. In *Arabidopsis*, the T-DNA mutants of *UGPase 1*, *UGPase 2* and double knockouts had no effect on growth rate, biomass production and flowering in mature plants, however, it was noted that young plantlets in agar had reduced hypocotyl and root length [25]. A small increase in plant height without apparent effect on dry weight was reported for tobacco plants overexpressing the *Acetobacter UGPase* under control of a constitutively expressed 35S promoter from cauliflower mosaic virus or a vascular-specific parsley 4-coumarate:CoA ligase (4CL) promoter [14]. In *Populus* plants overexpressing the same bacterial *UGPase* under the control of a constitutively expressed 35S promoter, however, was reported to display significant reduction in plant height, stem diameter, leaf area and internode length [15]. Contrastingly, we have observed that the leaf area is dramatically greater in *PdUGPase2* plants relative to control. The distinct aspect of the present work relative to the previous transgenic *Populus* UGPase study [15] is the use of a native gene from *Populus deltoides* here. Given the fact that PdUGPase2 has only a 20% amino acid similarity with that of *Acetobacter* UGPase (Additional file 1), a difference between phenotypic responses of plants overexpressing the two genes is not unexpected.

### Cell wall composition of *PdUGPase2* overexpressing *Populus*

**Figure 5:**
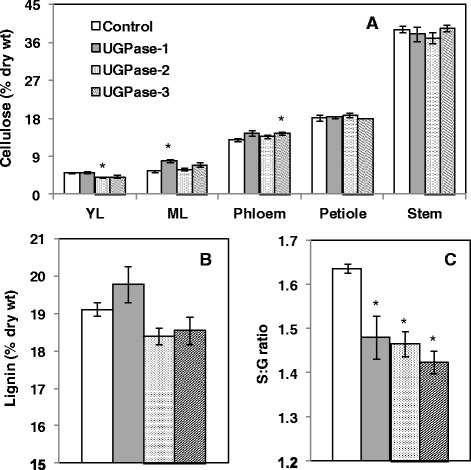
**Cell wall composition analysis in control and three** ***UGPase2*** **overexpression lines. A**. Cellulose levels in different tissues, **B**. Lignin and **C**. syringyl (S) to guaiacyl (G) ratio in stems of control and transgenic lines. YL and ML represent young and mature leaf, respectively. Data represent means ± SE (n ≥ 3). *indicates statistically significant, *p* ≤ 0.05 based on Student’s *t*-tests.

**Figure 6:**
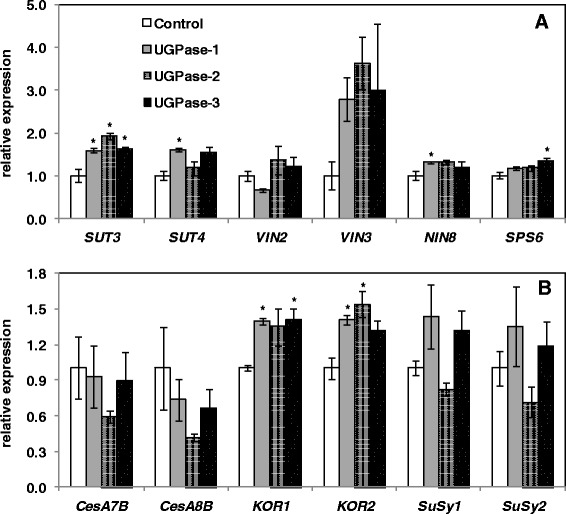
**Expression of selected sugar metabolism (A) and cellulose pathway genes (B) in young leaves of control and** ***UGPase2*** **overexpression lines.** Relative expression was calculated based on the expression of reference genes *Ubiquitin-conjugating enzyme E2* and *18S ribosomal RNA. SUT*, Sucrose transporter; *VIN*, Vacuolar invertase; NIN, Neutral invertase; *SPS*, Sucrose phosphate synthase; *CesA*, Cellulose synthase; *SuSy*, Sucrose synthase; *KOR*, KORRIGAN. Accessions: SUT3, Potri.019G085800; SUT4, Potri.002G106900; VIN2, Potri.003G112600; VIN3, Potri.015G127100; NIN8, Potri.019G082000; SPS6, Potri.018G124700; CesA7b, Potri.018G103900; CesA8b, Potri.004G059600; KOR1, Poptri.003G151700; KOR2, Poptri.001G078900; SuSy1, Potri.018G063500; SuSy2, Potri.006G136700;18S, AF206999; UBCc, Potri.006G205700. Data represent means ± SE (n = 3). *indicates statistically significant, *p* ≤ 0.05 based on Student’s *t*-tests.

HPLC analysis of stem cell wall sugar composition showed that glucose and xylose were the most abundant sugars in all samples as expected (Table 1). Among glucose, xylose, galactose, arabinose and mannose, significant changes were observed only in mannose levels, with lower mannose levels in transgenic samples. While cellulose and lignin content appeared unaffected; the syringyl-to-guaiacyl (S/G) ratio of lignin was significantly reduced in transgenic lines (Figure 5A - C). In order to explore whether overexpression of *UGPase2* has any effect on the transcription of carbohydrate metabolism or/and cellulose pathway, a subset of sugar metabolism and cellulose synthesis-related genes were studied (Figure 6). The panel of genes that we studied included, *sucrose transporters*, *SUT3*, linked previously to enhanced cellulose production [26], and *SUT4*, linked to carbon partitioning [27]; genes that encode sucrose hydrolyzing enzymes [1], *vacuolar invertases*, *VIN2* and *VIN3*; *neutral invertase*, *NIN8* [27]. Additionally, the secondary cell wall associated cellulose synthase (*CesA*) genes, *CesA7B* and *CesA8B*, and *KORRIGAN* (*KOR*) genes, *KOR1* and *KOR2*, were included based on previously reports of proposed roles in cellulose biosynthesis [28-30]. *SuSy1 and Susy2* transcripts and corresponding proteins have been shown to be elevated in tissue contexts with enhanced cellulose biosynthesis such as secondary xylem development and tension stress response [31,32]. Furthermore, a functional role for SuSy in supplying sugars for cellulose synthesis has been proposed [33]. The expression of most sugar metabolism genes was not significantly altered except *SUT3* which was significantly higher in all the three transgenic lines (Figure 6A). Whether SUT3 has a direct role in sucrose transport and carbon partitioning in *Populus* is as yet unanswered. Of the surveyed cellulose pathway genes, *CesA* and *SuSy* isoforms were not significantly altered, but *KOR1* and *KOR2* were significantly increased in young leaves of transgenic lines (Figure 6B). These results suggest that overexpression of *PdUGPase2* may be associated with modest changes in transcript levels of cellulose pathway-associated genes but that does not translate into changes in cellulose levels (Figure 5A). The unchanged cellulose levels in overexpression lines of *PdUGPase2* contradicts with previous reports where cellulose levels were increased in *AxUGPase* overexpression lines [15]. While the implication of low sequence identity between the bacterial and *Populus* UGPase isoforms on enzyme activity is yet to be clarified, the functional activity of *AxUGPase* has shown to complement cellulose negative mutant phenotype by channeling sugar substrate for cellulose synthesis [34].

**Table 1:** **Structural carbohydrate content (mg g** ^**−1**^ **) in stems of control and three** ***UGPase2*** **transgenic lines**

	**Cellobiose**	**Glucose**	**Xylose**	**Galactose**	**Arabinose**	**Mannose**
**Control**	3.54 ± 0.06	35.1 ± 0.62	15.6 ± 0.17	1.69 ± 0.04	0.92 ± 0.02	1.93 ± 0.05
**UGPase-1**	3.44 ± 0.17	34.1 ± 0.85	15.6 ± 0.18	1.62 ± 0.08	0.95 ± 0.05	1.36^*^ ± 0.06
**UGPase-2**	3.67 ± 0.14	34.1 ± 0.60	16.1 ± 0.23	1.28 ± 0.30	0.99 ± 0.03	1.59^*^ ± 0.07
**UGPase-3**	3.44 ± 0.12	33.3 ± 0.82	15.8 ± 0.24	1.75 ± 0.10	0.97 ± 0.05	1.49^*^ ± 0.06

Data represent means ± SE (n ≥ 3). ^*^indicates statistically significant, *p* ≤ 0.05 based on Student’s *t*-tests.

### Metabolic profiles of xylem, phloem and leaves of *PdGPase2* overexpressing *Populus*

Secondary metabolites such as phenolics and tannins are derived from the carbon in glucose channeled via the shikimate and phenylpropanoid pathway [4]. In order to characterize the broader changes in secondary metabolism, we undertook a detailed metabolomics study. As expected, the xylem tissue sample, which contain dead xylem cells, had the fewest statistically significant metabolite differences (p ≤ 0.05) between *PdUGPase2* overexpression lines and control plants (Additional file 5). Still, among the significant differences, shikimic acid and maleic acid were reduced to 55% and 26% of controls, respectively. Several amino acids [i.e., asparagine, glutamine, aspartic acid, γ-amino-butyric acid and 5-oxo-proline] and phenolic glycosides [i.e., including salicylic acid and its glucoside, salicylic acid-2-*O*-glucoside, 2, 5-dihydroxybenzoic acid-5-*O*-glucoside, 2-methoxyhydroquinone-1-*O*-glucoside, 2-methoxyhydroquinone-4-*O*-glucoside, salicin, a partially identified gallic acid ester of a dihydroxybenzoic acid glycoside at retention time (RT) 15.83 min, and an unidentified phenolic glycoside at RT 14.99 min, m/z 284, 269] were increased 1.4x-3.0x in the transgenics. Additionally, glycerol-1/3-phosphate and α-monopalmitin, two fatty acid-related metabolites, were moderately elevated 1.2-1.3x.

We have observed a significant increase (68%) in total phenolic levels in transgenic samples relative to control (Additional file 5). The increase in phenolics may be correlated with the decreased plant height and altered lignin composition. Down-regulation of *Cinnamoyl-CoA reductase* (*CCR*), a gene in monolignol pathway in *Populus* was reported to affect plant height, lignin content, S/G ratio, and reduction in several sugars including mannose, however, the levels of phenolics were found to be elevated [35]. Antisense down-regulation of *4CL* gene in *Populus* has been reported to result in reduced lignin content and plant height but increased levels of phenolics [36]. In the *PdUGPase* plants studies here, we observe a significant reduction in plant height and S/G ratio, and an increase in phenolics.

Many more metabolites were affected in the phloem of *UGPase2* transgenic lines relative to control plants (Additional file 6). Similar responses included the reduction of shikimic acid and increases in 2, 5-dihydroxybenzoic acid-5-*O*-glucoside, salicylic acid-2-*O*-glucoside and salicin. An example of a contrasting response included the decline in the phenolic acid glycoside (RT 14.58, m/z 284, 269) that was elevated in the xylem of *UGPase2* plants. Additionally, there were declines in soluble sugars in the phloem of *UGPase2* lines, including decreases in fructose, galactose, glucose and raffinose ranging from 40-68%, compared to that of controls. Many aromatic metabolites were reduced, including catechin, catechol, salicyl alcohol, salicylic acid and higher-order salicylates, including salicortin and α-salicyloylsalicin and five caffeoyl-conjugates, two of which (eluting at RT 20.69 and 20.77 min) were not detected in plants overexpressing *UGPase2* (Additional file 7). These latter declines were coupled with a decline in sinapyl alcohol and its glucoside, syringin, but not coniferyl alcohol nor its glucoside, coniferin. These observations sit in contrast with the large number of increases in caffeic acid and other caffeoyl-conjugates and feruloyl-conjugates in the phloem of *UGPase2* transgenic lines. Overall, both the total phenolic metabolites and soluble sugars were reduced in the phloem of *UGPase2* lines relative to the levels measured in xylem.

UDP-glucose acts as a substrate for glycosylation of small secondary metabolites [37,38]. The effect of *UGPase2* overexpression on the ratio of aglycones to glycosides of phenolics was measured in phloem samples of control and transgenic plants (Additional file 8). We found that as compared to controls, *UGPase2* transgenic plants have a greater glycoside to aglycone ratio for the salicylates and a few other aromatic acids, but not for the monolignol glycosides. The most prominent change was a 5-fold increase in salicin, a possible precursor of higher-order phenolic glycosides.

**Figure 7:**
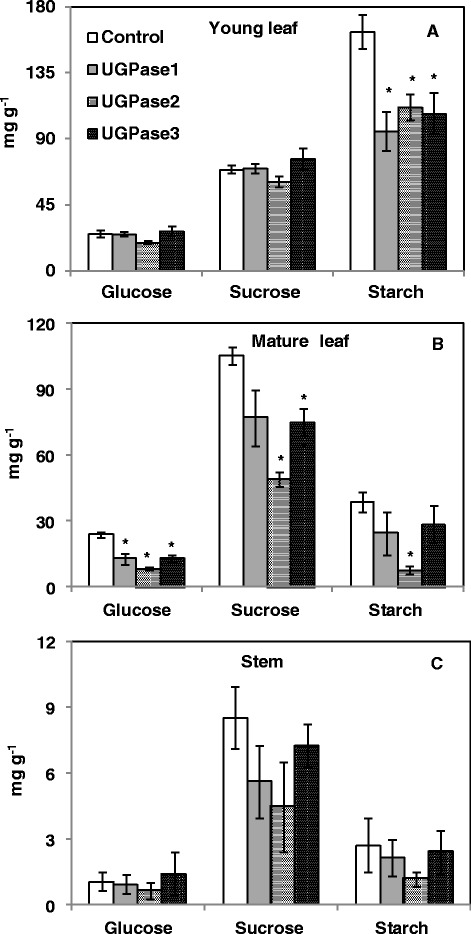
**Estimation of non-structural carbohydrates in control and** ***UGPase2*** **transgenic lines.** Levels of glucose, sucrose and starch in young leaves **(A)**, mature leaves **(B)** and stems **(C)** are presented. Data represent means ± SE (n ≥ 3). * indicates statistically significant, *p* ≤ 0.05 based on Student’s *t*-tests.

In general, metabolomic responses in leaves of *PdUGPase2* overexpression lines were similar to those in the phloem (Additional files 9, 10). In contrast with stem phloem responses, caffeoylpopuloside and a caffeoyl-conjugate at RT 19.57 declined. Additionally, *cis*- and *trans*-3-*O*-caffeoylquinic acids were enhanced in leaves, as were three other caffeoyl-conjugates (RT 19.14, 19.89 18.14). Two caffeoyl-glycoside conjugates at RT 19.14 and 19.31 were not detectable in leaves of control plants (Additional file 10). Other benzoic acid-related and salicylic acid-related metabolites in the transgenics, including 2,3-dihydroxybenzoic acid-3-*O*-glucoside, a partially identified dihydroxybenzoic acid-gallic acid glycoside (RT 15.83), salicyl alcohol, catechol, and salicyloyl-salicortin, were higher. Reductions were observed in fatty acid-related metabolites, including glyceric acid and, mono- and di-galactosylglycerol. Neither salicylic acid nor its glucoside were significantly different between in leaves of transgenic and control plants. Furthermore, calorimetric assays showed that phenolic content was higher, and tannin, starch, sucrose, and glucose levels were lower in transgenic leaves relative to control (Figure 7, Table 2).

**Table 2:** **Levels of phenolics and tannins from mature leaves and petiole of control and three** ***UGPase2*** **overexpression lines**

	**Leaf**		**Petiole**	
	**Phenolics mg g** ^**−1**^	**Tannins OD g** ^**−1**^	**Phenolics mg g** ^**−1**^	**Tannins OD g** ^**−1**^
**Control**	130.0 ± 5.0	30.5 ± 3.1	114.5 ± 4.2	73.2 ± 4.7
**UGPase-1**	141.2 ± 6.7	22.4 ± 2.8	65.3^*^ ± 6.0	20.5^*^ ± 5.7
**UGPase-2**	156.1* ± 7.7	21.3 ± 6.3	73.0^*^ ± 3.6	16.6^*^ ± 3.5
**UGPase-3**	150.3 ± 13.0	17.1* ± 1.3	72.6* ± 5.9	20.9^*^ ± 2.2

Data represent means ± SE (n ≥ 3). *indicates statistically significant, *p* ≤ 0.05 based on Student’s *t*-tests.

### Similar metabolite phenotypes of *Populus* plants misexpressing two distinct candidate cell wall biosynthesis genes

It is interesting to note that many of the metabolic responses reported here were similarly observed in a recent study of *P. deltoides KOR*-*like* RNAi knockdown mutants (Payyavula et al. in review). That is, with both overexpression of *PdUGPase2* in the present study and knockdown of *KOR*-*like* genes, there was an accumulation of caffeoyl-conjugates, including caffeoylpopuloside. Both types of transgenics also had declines in the same types of caffeoyl-conjugates.

The close parallel in the metabolic phenotypes between these different transgenics was also evident in the leaves, where both exhibited declines in syringin and caffeoyl conjugates at RT 20.69 and 20.31 min and increases in *cis*- and *trans*-3-*O*-caffeoylquinic acid and caffeoyl-conjugates at RT 19.14, 19.31, 18.14, 21.52, 20.92, 19.89, 19.53, 18.24 min. Given that these two groups of genes have been proposed to play a role in cell wall biosynthesis and remodeling pathways [29,39,40], the premise of observed similarities in metabolite levels in response to perturbations of cell wall formation is interesting.

With S-lignin deposition affected more than G-lignin, there may be a reduced flux of carbon to lignin precursors at the terminus of the lignin pathway. This hypothesis is supported by accumulation of phenolic acid (caffeoyl and feruloyl) conjugates upstream of monolignol synthesis with shikimic acid and other unidentified moieties (Additional files 5, 6 and 7). In *CCR* down-regulated *Populus,* the decrease in lignin and S/G ratio was accompanied by an increase in feruloyl conjugates [35]. A thorough characterization of accumulating caffeoyl-conjugates is merited, likely revealing the nature of lignin or cell wall storage components that have not previously been documented. The greater number and nature of metabolite responses in phloem versus xylem suggests that metabolite trafficking between these organs plays a major role in shuttling, modifying and storing precursors that are directly not incorporated into the cell wall. Therefore, metabolite trafficking mechanisms in cell wall assembly also merit more attention. Additional detailed study of alterations in cell wall-related properties such as cellulose crystallinity, lignin-carbohydrate cross-linkages and structural features revealed by electron microscopy, as well as stem strength properties are needed to support a possible explanation of the observed similarity in metabolite profiles as a potential consequence of the aberrant cell wall formation in *PdUGPase* overexpression and *PdKOR knockdown* transgenic plants.

### Overexpression of a bacterial or *Populus UGPase* result in contrasting phenotypes

In tobacco, overexpression of *AxUGPase* under the control of a cauliflower mosaic virus *35S* promoter or a vascular-specific parsley 4CL promoter resulted in a slight increase in plant height via increased internode length [14]. However, total plant dry weight was unaltered. Although overexpression of the *AxUGPase* was evident at the transcript level, in both leaves and xylem, UGPase enzyme activity was not significantly altered. In contrast, overexpression of the same *AxUGPase* under the control of a *35S* promoter in hybrid poplar resulted in significant reduction in plant height, stem diameter, leaf area and internode length [15]. While sucrose and glucose levels were greater in hybrid poplar overexpressing *AxUGPase* [15], their levels were lower in *Populus* overexpressing the native *UGPase2* under the control of a Ubiquitin promoter. Moreover, leaf area of transgenic hybrid poplar plants overexpressing *AxUGPase* were nearly 75% smaller relative to control, while *Populus* plants overexpressing native *UGPase2* resulted in increased leaf area by a factor of two (Figure 3D). One plausible explanation is that the differential conserved domain composition of AxUGPase and PdUGPase2 enzymes (Additional file 1) contributes to differential utilization of sugars synthesized in transgenic leaves towards either leaf growth or storage purposes.

At the metabolic level, our results also contrast with those reported for hybrid poplar overexpressing a bacterial *UGPase*, such that there was a 233x increase in salicylic acid-2-*O*-glucoside in developing xylem, relatively no change in other phenolic glycosides and elevated soluble sugars using the *AxUGPase* construct [15]. In our study, salicylic acid-2-*O*-glucoside was only modestly elevated (2.70x) in xylem, along with a number of other phenolic glycosides with a similar magnitude of response of 1.39-2.65x. The greatest number and magnitude of metabolite responses were observed in phloem tissue of the stem, where both soluble sugars and total phenolic metabolites declined (Additional file 6). A decline in higher-order salicylates was offset by large accumulations of conjugates of caffeic acid and ferulic acid. In contrast to the study undertaken by Coleman et al. [15], where lignin content was reported to be reduced and the S/G ratio increased, transgenic stem lignin content in the present study was unchanged while S/G ratio decreased. This change in lignin composition (decrease in S units of lignin) co-occurred with an increase in the concentrations of monolignols and their glucosides in the phloem of stems. While coniferyl alcohol and its glucoside, coniferin were unaltered in phloem of *UGPase2* plants, sinapyl alcohol and its glucoside, syringin, were both greatly reduced.

## Conclusion

The present report addresses a gap in our understanding of the functional role of *UGPase2* in woody perennials such as *Populus*. We have presented metabolomics-based evidence in support of a functional role for a plant *UGPase2* in the tightly coupled primary and secondary metabolic pathways. Our results are in contrast with previously reported results based on overexpression of a low-identity bacterial *UGPase* in hybrid poplar. The observed lack of impact on cellulose levels and contrasting plant morphology of *Populus UGPase2* overexpression lines lead to the hypothesis that plant *UGPases* may have evolved to play a functional role divergent from that reported for bacterial *UGPase*. Future research aimed at clarifying the precise enzymatic roles and pathway contexts of *UGPase2* would provide mechanistic insights into the pronounced phenotypic effects resulting from overexpression of a native *UGPase2* in *Populus*.

## Methods

### Sequence and phylogenetic analyses

Protein sequence information for *Populus* was collected from *Phytozome v9.1* [http://www.phytozome.net/cgi-bin/gbrowse/Populus/]: *Populus trichocarpa v3.0* [20,41]. Other sequence information (e.g. nucleotide sequence of genes) was obtained from NCBI database. The amino acid sequences were aligned using ClustalW program and phylogenetic tree was developed using MEGA (Molecular Evolutionary Genetics Analysis) software with neighbor joining method using 500 independent bootstrap runs [42]. Protein sequence similarity percentage was calculated with GeneDoc program [43].

### Plant materials

An overexpression construct was developed by cloning the full-length coding sequence of *PdUGPase2* (Potri.017G144700, also referred to in the present work as *PtrUGPase2* or *UGPase2*) from *Populus deltoides* under the control of Ubiquitin 3 promoter (Accession L05363) in the pAGW560 binary vector. Agrobacterium-based transformation was performed at ArborGen LLC, Ridgeville, SC. Transgenics, along with empty vector control plants (also referred to in this report as control or as control plants or lines), were propagated at 25°C and 16 h day light in greenhouses at Oak Ridge National Laboratory, Oak Ridge, TN. Plant propagation was performed using greenwood stem cuttings. Initially, stem cuttings were propagated in small leach tubes and grown to a plant height of approximately 50 cm, following which, the plants were moved to larger pots and grown under automated drip irrigation and fertilization systems. In our preliminary studies, we included ten independent transgenic lines plus eleven empty vector controls. Further in-depth studies were performed on up to three selected lines. At the time of harvest, plant height was measured from shoot tip to stem base (region of stem 6 cm above the soil surface). Stem diameter was measured at the base using calipers. Approximate leaf area was calculated by multiplying midrib length by maximum width, and averaging the estimates of five individual leaves between LPI-1(leaf plastochron index 1) to LPI-5 as described previously [27]. Plants were harvested between 12:00 PM and 2:00 PM. At the time of harvest, young leaf (LPI 1), mature leaf (LPI-6), young stem (internodes 1 to 3), mature stem (internodes 6 to 8), petiole of mature leaf, phloem (bark), xylem (stem scrapings) and root were collected. Immediately after harvest, phloem samples were frozen in dry ice, stem samples were air-dried, and the rest were frozen in liquid nitrogen and stored at −80°C until used. For root architecture scans, plants growing in leach tubes were destructively harvested and scanned using Epson Perfection V700 photo scanner (Epson America Inc, Long Beach, CA), and root surface area was estimated using WinRhizo (Version 2012b) software.

### Non-structural carbohydrate analysis

Levels of sucrose and glucose were estimated as described previously [44] with slight modifications. Such that, ca. 20 mg of freeze-dried sample was extracted thrice using a total of 2.0 ml of 80% ethanol by incubating at 80°C. The supernatants from the three extracts were combined and again extracted with 50 mg activated charcoal to eliminate pigments. After centrifugation, the remaining pellet was used for starch analysis and supernatant for sugar estimation. The ethanol phase from the supernatant was eliminated by incubating the sample at 50°C for 18 hr. The pellet was dissolved in water (800 μl for leaf and 250 μl for stem sample), vortexed and used for sucrose and glucose estimation using kits (Sigma, St Louis, MO). Starch from the pellet was estimated using enzymatic method and quantified using glucose standard as described previously [27,45].

### Structural carbohydrate analysis

Structural carbohydrates were extracted from the dried pellet after sugars and starch extraction. Briefly, 5 mg of the extract-free sample was digested with H_2_SO_4_ (50 μl of 72% v:v) at 37°C for 60 min. After diluting to a final concentration of 4% H_2_SO_4_, the samples were autoclaved for 60 min, cooled to room temperature and an aliquot was neutralized with CaCO_3._ Carbohydrate composition was estimated using high performance liquid chromatography (LaChrom Elite® system, Hitachi High Technologies America, Inc.) equipped with a refractive index detector (model L-2490) and a UV–Vis detector (model L-2420) as described elsewhere [46,47].

### RNA extraction and qRT-PCR

RNA was extracted using the Spectrum Plant Total RNA Kit (Sigma) with slight modification. To approximately 50 mg frozen sample, 850 μl of CTAB (cetyl trimethylammonium bromide) buffer (pre-warmed at 65°C) and 10 μl of β-mercaptoethanol (Sigma) was added, vortexed and incubated at 65°C for 5 min. To the same tube 600 μl of chloroform:isoamyl alcohol (24:1 v:v) was added and re-extracted. After centrifugation, the supernatant was passed through filter column (Sigma). After diluting the filtrate with 500 μl of 95% EtOH, the combined liquid was passed through binding column. The binding column was then washed with Wash 1 solution and on-column DNAse digestion was carried out for 20 min as per the manufactures instructions (Sigma). The binding column was washed with wash buffers and RNA was eluted using 50–100 μl elution buffer. After quantifying RNA using NanoDrop, cDNA was prepared from 1500 ng total RNA. qRT-PCR was performed using 3 ng RNA equivalent cDNA, gene specific primers (200 nM) (primer sequences provided in Additional file 11) [48] and 1Z SYBR green (Bio-Rad life sciences, Hercules, CA) on the StepOnePlusTM Real-Time PCR system (Applied Biosystems, Grand Island, NY). Gene expression was calculated using ΔcT or ΔΔcT methods [49] based on the threshold cycle (cT) values of the target gene and the housekeeping genes, *Ubiquitin-conjugating enzyme E2* (Potri.006G205700) and *18S ribosomal RNA* (AF206999) (Additional file 11)*.*

### Phenolics and pigment analysis

Freeze-dried sample (5 mg) was extracted twice with a total of 1.5 ml 80% EtOH using TissueLyzer (Qiagen, Valencia, CA) for 5 min at 30 Hz. After 5 min centrifugation, the resulting supernatant was used for estimation of total phenolics, and pellet for tannin analysis. Total phenolics was estimated by using Folin–Ciocalteu reagent as described previously [50] with modification. In a 96-well plate, 10 μl of extract, 150 μl water and 10 μl FC reagent (Sigma) were added, mixed. After 3 min incubation, 30 μl of 20% sodium carbonate was added and again incubated at 40°C. After 30 min, absorbance at A_755_ was measured using a plate reader (SpectraMax, Molecular Devices, Sunnyvale, CA) and phenolic content was estimated based on the standard curve developed using chlorogenic acid. Tannins from the pellet were estimated using butanol:HCl (95:5 v:v) method as described elsewhere [51].

### Cellulose content

The level of total cellulose content was estimated by the anthrone reagent method as described previously [52]. Sample (roughly 25 mg air-dried and milled stem sample or 15 mg freeze-dried leaf, phloem or petiole) was first digested with 500 μl acetic-nitric reagent (10% nitric acid in 80% acetic acid, v:v) at 98°C for 30 min, followed by washing the pellet with 700 μl water. After complete removal of water, the pellet was digested with 600 μl of 67% H_2_SO_4_ for 60 min. After a 10-fold dilution with water, 10 to 50 μl of diluted sample along with water to a final volume of 50 μl were taken in 0.2 ml PCR tube, added 100 μl anthrone solution (0.5 mg ml^−1^ in H_2_SO_4_) and incubated at 98°C for 10 min in a thermal cycler. After cooling, the samples were transferred to a 96-well plate and absorbance at A_630_ was recorded and cellulose content was estimated based on the standard curve developed using glucose dilutions.

### Lignin content and its composition analysis

Lignin and the ratio of its syringyl-to-guaiacyl units (S/G ratio) from air dried stem samples (4 mg) were analyzed by pyrolysis molecular beam mass spectrometry (MBMS) at National Renewable Energy Laboratory, Golden, CO as described elsewhere [53].

### Metabolite profiling

Individual metabolites were analyzed by metabolite profiling using gas chromatography–mass spectrometry (GC-MS). Briefly, 50–75 mg of finely ground fresh tissue sample were repeatedly extracted with 2.5 ml of 80% ethanol, with the extracts then combined. A 1 ml aliquot was dried in a nitrogen stream. After dissolving the dried extracts in acetonitrile followed by trimethylsilylation, metabolite profiling was performed by GC-MS, as described elsewhere [54,55]. Metabolites were identified based on mass spectral fragmentation patterns of electron impact ionization (70eV) and were quantified using peak areas of a characteristic mass-to-charge (m/z) ratio normalized to the internal standard (sorbitol) recovered and corrected for sample wieght. Some of the unidentified metabolites were denoted by their retention time (RT) and key m/z ratio. The metabolite data were presented as fold changes of the transgenic line (average of 3 independent lines with 3 replicates for each line) vs. the average of the controls. Statistical significance was assessed using Student’s *t*-test.

### Availability of supporting data

All the data supporting our results are included in the article and in the Additional files.

## Additional files

Additional file 1:
**Protein sequence similarity/identity percentage among selected UGPases.**
Additional file 2:
**Protein sequence alignment of the two** ***PdUGPase*** **isoforms with that of previously characterized members.**
Additional file 3:
**Phenotypic characterization of** ***UGPase2*** **transgenic plants in preliminary study.**
Additional file 4:
**Relative expression of** ***UGPase2*** **in mature leaf petioles of control and the three transgenic lines.**
Additional file 5:
**Metabolite levels in xylem of control and** ***UGPase2*** **overexpression lines.**
Additional file 6:
**Metabolite levels in phloem of control and** ***UGPase2*** **overexpression lines.**
Additional file 7:
**Unknown caffeoyl-glycosides in** ***P. deltoides*** **stem phloem of control plants but absent in overexpressed** ***UGPase2*** **plants.**
Additional file 8:
**Ratio of glycosides to aglycones of phenolics in phloem of control and** ***UGPase2*** **overexpression lines.**
Additional file 9:
**Metabolite levels in mature leaf of control and** ***UGPase2*** **overexpression lines.**
Additional file 10:
**Unknown caffeoyl-glycosides in** ***P. deltoides*** **leaves unique to overexpressed** ***UGPase2*** **transgenic lines.**
Additional file 11:
**List of gene primer sequences and gene models used in this study.**

## Abbreviations

ADP: Adenosine 5ˈ-diphosphate
ATP: Adenosine triphosphate
4CL: 4-coumarate:CoA ligase
CCR: Cinnamoyl-CoA reductase
CesA: Cellulose synthase
cT: Threshold cycle
CTAB: Cetyl trimethylammonium bromide
GC-MS: Gas chromatography–mass spectrometry
HPLC: High performance liquid chromatography
I-loop: Insertion loop
LPI: Leaf plastochron index
KOR: KORRIGAN
MBMS: Molecular beam mass spectrometry
MEGA: Molecular evolutionary genetics analysis
NB-loop: Nucleotide binding loop
NIN: neutral invertase
qRT-PCR: Quantitative reverse transcriptase PCR
RNAi: RNA interference
RT: Retention time
S/G: Syringyl to guaiacyl ratio
SPS: Sucrose phosphate synthase
SuSy: Sucrose synthase
SUT: sucrose transporter
UDP: Uridine 5ˈ-diphosphate
UTP: *Uridine triphosphate*
UGPase: UDP-glucose pyrophosphorylase
VIN: Vacuolar invertase
