# Supplementary material for: Metabolic profiling reveals altered sugar and secondary metabolism in response to UGPase overexpression in Populus
Source: BMC Plant Biol. 2014 Oct 7;14:265. doi: 10.1186/s12870-014-0265-8 (PMC4197241; doi:10.1186/s12870-014-0265-8)
Supplement: Additional file 1: — Protein sequence similarity/identity percentage among selected UGPases. [file 12870_2014_265_MOESM1_ESM.doc]

Additional file 1. Protein sequence similarity/identity percentage among selected UGPases. Accession numbers are as presented in Fig. 1.
