# Supplementary material for: Metabolic profiling reveals altered sugar and secondary metabolism in response to UGPase overexpression in Populus
Source: BMC Plant Biol. 2014 Oct 7;14:265. doi: 10.1186/s12870-014-0265-8 (PMC4197241; doi:10.1186/s12870-014-0265-8)
Supplement: Additional file 2: — Protein sequence alignment of the two PdUGPase isoforms with that of previously characterized members. [file 12870_2014_265_MOESM2_ESM.doc]

**Additional file 2.** Protein sequence alignment of the two PdUGPase isoforms with that of previously characterized members. NB-loop and I loop represent nucleotide binding loop and insertion loop, respectively. Accessions are as presented in Fig. 1.
