# Supplementary material for: Metabolic profiling reveals altered sugar and secondary metabolism in response to UGPase overexpression in Populus
Source: BMC Plant Biol. 2014 Oct 7;14:265. doi: 10.1186/s12870-014-0265-8 (PMC4197241; doi:10.1186/s12870-014-0265-8)
Supplement: Additional file 3: — Phenotypic characterization of UGPase2 transgenic plants in preliminary study. [file 12870_2014_265_MOESM3_ESM.doc]

**Additional file 3.** Phenotypic characterization of *UGPase2* transgenic plants in preliminary study. Plant height (A) and stem diameter (B) of control and *UGPase2* overexpressed transgenic lines*.* Data represent means ± SE (n ≥ 3). * indicates statistically significant, *p* < 0.05 based on Student’s *t*-tests.
