# Supplementary material for: Metabolic profiling reveals altered sugar and secondary metabolism in response to UGPase overexpression in Populus
Source: BMC Plant Biol. 2014 Oct 7;14:265. doi: 10.1186/s12870-014-0265-8 (PMC4197241; doi:10.1186/s12870-014-0265-8)
Supplement: Additional file 4: — Relative expression of UGPase2 in mature leaf petioles of control and the three transgenic lines. [file 12870_2014_265_MOESM4_ESM.doc]

**Additional file 4.** Relative expression of *UGPase2* in mature leaf petiole of control and three transgenic lines. Relative expression was calculated based on the expression of reference genes *Ubiquitin-conjugating enzyme E2* (Potri.006G205700) and *18S ribosomal RNA* (AF206999)*.* Data represent means ± SE (n ≥ 3). * indicates statistically significant, *p* < 0.05 based on Student’s *t*-tests.
