# Supplementary material for: Metabolic profiling reveals altered sugar and secondary metabolism in response to UGPase overexpression in Populus
Source: BMC Plant Biol. 2014 Oct 7;14:265. doi: 10.1186/s12870-014-0265-8 (PMC4197241; doi:10.1186/s12870-014-0265-8)
Supplement: Additional file 5: — Metabolite levels in xylem of control and UGPase2 overexpression lines. [file 12870_2014_265_MOESM5_ESM.doc]

| Xylem | Control | | UGPase | | Fold change |  |
| --- | --- | --- | --- | --- | --- | --- |
| Metabolite | Mean | sem | Mean | sem | UGPase/ Control | P-value |
| putrescine | 6.3 | 1.6 | 23.0 | 10.8 | 3.63 | 0.101 |
| asparagine | 215 | 31 | 644 | 155 | 2.99 | 0.006 |
| salicylic acid-2-O-glucoside | 1.3 | 0.1 | 3.5 | 0.6 | 2.7 | 0.001 |
| N-metabolite (11.98; 232, 449, 359) | 27 | 5 | 73 | 17 | 2.68 | 0.009 |
| dihydroxybenzoic acid-galloyl-glycoside (15.83) | 1.1 | 0.1 | 2.8 | 0.3 | 2.65 | 0.000 |
| 10.03; 159 116 | 101 | 15 | 250 | 56 | 2.47 | 0.009 |
| 2,5-dihydroxybenzoic acid-5-O-glucoside | 21 | 3 | 49 | 7 | 2.29 | 0.001 |
| glycoside (13.97; 171, 289) | 26 | 2 | 59 | 4 | 2.24 | 0.000 |
| glutamine | 685 | 82 | 1373 | 174 | 2.00 | 0.001 |
| salicylic acid | 86 | 18 | 155 | 20 | 1.79 | 0.016 |
| 2-methoxyhydroquinone-1-O-glucoside | 32 | 2 | 53 | 4 | 1.67 | 0.000 |
| salicin | 230 | 24 | 377 | 61 | 1.64 | 0.022 |
| aspartic acid | 289 | 27 | 451 | 86 | 1.56 | 0.059 |
| γ-amino-butyric acid | 649 | 48 | 1008 | 209 | 1.55 | 0.075 |
| phenolic glycoside (14.99; 284, 269) | 9.4 | 0.6 | 14.6 | 1.0 | 1.55 | 0.000 |
| 5- oxo-proline | 656 | 45 | 916 | 126 | 1.40 | 0.045 |
| 2-methoxyhydroquinone-4-O-glucoside | 11 | 1 | 16 | 1 | 1.39 | 0.002 |
| glycerol-1/3-phosphate | 20 | 1 | 26 | 2 | 1.29 | 0.012 |
| 10.61; 306, 288, 173, M+378 | 11 | 1 | 14 | 2 | 1.27 | 0.187 |
| cis-aconitic acid | 2.1 | 0.3 | 2.6 | 0.3 | 1.21 | 0.319 |
| glutamic acid | 138 | 14 | 167 | 24 | 1.21 | 0.288 |
| glucose | 1829 | 367 | 2188 | 633 | 1.20 | 0.612 |
| a-monopalmitin | 34 | 1 | 41 | 3 | 1.19 | 0.049 |
| myoinositol | 1288 | 162 | 1514 | 280 | 1.18 | 0.471 |
| galactose | 198 | 41 | 226 | 68 | 1.14 | 0.716 |
| serine | 74 | 11 | 83 | 16 | 1.12 | 0.648 |
| sucrose | 11490 | 808 | 12335 | 1228 | 1.07 | 0.557 |
| 1-linoleyl-rac-glycerol | 4.9 | 0.4 | 5.2 | 0.5 | 1.06 | 0.625 |
| valine | 171 | 38 | 152 | 40 | 0.89 | 0.732 |
| isoleucine | 50 | 13 | 44 | 16 | 0.88 | 0.774 |
| raffinose | 31 | 6 | 27 | 6 | 0.86 | 0.607 |
| phenylalanine | 13 | 2 | 10 | 1 | 0.77 | 0.320 |
| threonine | 102 | 21 | 74 | 18 | 0.72 | 0.318 |
| threonic acid | 1407 | 335 | 911 | 260 | 0.65 | 0.272 |
| 11.16; 218, 335 | 63 | 7 | 41 | 7 | 0.64 | 0.033 |
| 7.29; 86, 188 | 123 | 29 | 70 | 18 | 0.57 | 0.157 |
| shikimic acid | 373 | 47 | 204 | 31 | 0.55 | 0.009 |
| fructose | 1664 | 930 | 804 | 271 | 0.48 | 0.430 |
| maleic acid | 73 | 14 | 19 | 3 | 0.26 | 0.002 |

Additional file 5. Metabolite levels in xylem of control and *UGPase2* overexpression lines.

Mean and standard error of the mean (sem) metabolite concentrations (μg g^-1^ fresh weight in sorbitol equivalent response) from xylem tissue of overexpression *UGPase2* transgenic *Populus deltoides* and nontransgenic control plants. The fold change of the metabolite concentrations (average of 3 independent lines with 3 replicates for each line) of *UGPase2* versus control plants and the *P*-value of the contrast as determined by Student’s *t*-tests are shown
