# Supplementary material for: Metabolic profiling reveals altered sugar and secondary metabolism in response to UGPase overexpression in Populus
Source: BMC Plant Biol. 2014 Oct 7;14:265. doi: 10.1186/s12870-014-0265-8 (PMC4197241; doi:10.1186/s12870-014-0265-8)
Supplement: Additional file 6: — Metabolite levels in phloem of control and UGPase2 overexpression lines. [file 12870_2014_265_MOESM6_ESM.doc]

| Phloem | Control | | UGPase | | Fold change |  |
| --- | --- | --- | --- | --- | --- | --- |
| Metabolite | Mean | sem | Mean | sem | UGPase/ Control | P-value |
| feruloyl-conjugate (19.34; 171, 331 642, 627) | 6.2 | 0.5 | 53.6 | 2.9 | 8.67 | 0.000 |
| caffeoyl-shikimate (20.92; 463, 219) | 7.7 | 0.6 | 47.1 | 8.6 | 6.12 | 0.000 |
| caffeoyl-glycoside (19.31; 331, 171) | 6.7 | 0.8 | 31.1 | 2.2 | 4.63 | 0.000 |
| caffeoyl-shikimate (19.53; 169) | 3.8 | 0.3 | 16.4 | 1.1 | 4.27 | 0.000 |
| feruloyl-conjugate (18.14; 171) | 1.9 | 0.2 | 7.3 | 0.4 | 3.95 | 0.000 |
| caffeoyl-shikimate (18.24; 171, 219, 463) | 2.6 | 0.2 | 7.7 | 2.9 | 2.95 | 0.023 |
| salicin | 490 | 54 | 1327 | 232 | 2.71 | 0.000 |
| caffeoylpopuloside | 1.0 | 0.2 | 2.6 | 0.2 | 2.63 | 0.000 |
| caffeoyl-conjugate (15.35) | 0.8 | 0.2 | 1.8 | 0.3 | 2.42 | 0.001 |
| phenolic (23.7; 193, 271, 267, 355, 481, 571) | 46 | 7 | 110 | 18 | 2.36 | 0.000 |
| caffeoyl-conjugate (19.57; 171, 97, 208) | 4.2 | 0.2 | 8.9 | 0.5 | 2.11 | 0.000 |
| caffeoyl-glycoside (21.52; 271, 469, 219) | 3.4 | 0.3 | 5.9 | 0.4 | 1.75 | 0.000 |
| phenolic (23.29; 255, 193, 271, 355, 481, 571) | 53 | 4 | 89 | 9 | 1.67 | 0.000 |
| phenolic (23.53; 255, 193, 271, 481, 571) | 40 | 3 | 61 | 6 | 1.51 | 0.002 |
| salicylic acid-2-O-glucoside | 3.9 | 0.3 | 5.7 | 0.8 | 1.46 | 0.015 |
| 2,5-dihydroxybenzoic acid-5-O-glucoside | 68 | 4 | 94 | 9 | 1.38 | 0.006 |
| glycoside (13.91; 171, 289) | 2.0 | 0.1 | 2.6 | 0.3 | 1.34 | 0.011 |
| 6-hydroxy-2-cyclohexenone alcohol | 26 | 4 | 35 | 6 | 1.33 | 0.256 |
| phenolic (23.17; 255, 193, 271, 481, 571) | 118 | 10 | 150 | 14 | 1.28 | 0.056 |
| 6-hydroxy-2-cyclohexenone (enol) | 4.1 | 0.3 | 5.0 | 0.4 | 1.22 | 0.055 |
| caffeic acid | 23 | 1 | 28 | 2 | 1.20 | 0.044 |
| dihydroxybenzoic acid-galloyl-glycoside (15.83) | 5.2 | 0.3 | 6.1 | 0.6 | 1.16 | 0.175 |
| 3-O-caffeoylquinic acid | 3.4 | 0.3 | 3.7 | 0.4 | 1.08 | 0.578 |
| glyceric acid | 17 | 1 | 18 | 1 | 1.05 | 0.562 |
| salicyloyl-salicortin | 156 | 28 | 160 | 33 | 1.03 | 0.915 |
| coniferin | 0.6 | 0.1 | 0.6 | 0.1 | 1.02 | 0.949 |
| digalactosylglycerol | 14 | 1 | 13 | 1 | 0.97 | 0.824 |
| ferulic acid | 3.8 | 0.2 | 3.6 | 0.2 | 0.96 | 0.637 |
| 2,3-dihydroxybenzoic acid-3-O-glucoside | 61 | 3 | 58 | 4 | 0.94 | 0.511 |
| citric acid | 1863 | 144 | 1747 | 156 | 0.94 | 0.610 |
| sucrose | 14220 | 524 | 12886 | 627 | 0.91 | 0.122 |
| ethyl-phosphate | 333 | 15 | 300 | 23 | 0.90 | 0.219 |
| coniferyl alcohol | 0.7 | 0.0 | 0.6 | 0.0 | 0.89 | 0.269 |
| quinic acid | 40 | 2 | 34 | 3 | 0.86 | 0.170 |
| phenolic glycoside (17.23; 179) | 253 | 21 | 200 | 27 | 0.79 | 0.136 |
| salicylic acid | 817 | 32 | 616 | 38 | 0.75 | 0.000 |
| monogalactosylglycerol | 15 | 5 | 11 | 1 | 0.71 | 0.545 |
| catechol | 285 | 14 | 190 | 35 | 0.66 | 0.005 |
| 11.16; 218, 335 | 42 | 2 | 27 | 14 | 0.65 | 0.173 |
| phenolic glycoside (14.58; 284, 269) | 257 | 12 | 161 | 9 | 0.63 | 0.000 |
| fructose | 507 | 56 | 305 | 57 | 0.60 | 0.024 |
| galactose | 151 | 14 | 78 | 11 | 0.52 | 0.001 |
| α-salicyloylsalicin | 2492 | 220 | 1254 | 204 | 0.50 | 0.001 |
| catechin | 805 | 53 | 398 | 60 | 0.49 | 0.000 |
| sinapyl alcohol | 1.6 | 0.1 | 0.8 | 0.0 | 0.49 | 0.000 |
| caffeoyl-conjugate (20.4) | 4.1 | 0.2 | 2.0 | 0.6 | 0.48 | 0.000 |
| salicyl alcohol | 141 | 8 | 67 | 6 | 0.47 | 0.000 |
| shikimic acid | 195 | 14 | 92 | 10 | 0.47 | 0.000 |
| syringin | 5.0 | 0.3 | 2.3 | 0.2 | 0.47 | 0.000 |
| salicortin | 4569 | 254 | 2100 | 236 | 0.46 | 0.000 |
| 6-hydroxy-2-cyclohexenone-1-carboxylic acid | 174 | 15 | 78 | 6 | 0.45 | 0.000 |
| glucose | 1867 | 133 | 820 | 113 | 0.44 | 0.000 |
| raffinose | 137 | 22 | 44 | 7 | 0.32 | 0.004 |
| caffeoyl-conjugate (19.89; 463, 219, 255) | 34.9 | 2.2 | 9.3 | 0.7 | 0.27 | 0.000 |
| caffeoyl-conjugate (18.62; 171, 219) | 13.7 | 0.6 | 2.4 | 0.3 | 0.17 | 0.000 |
| caffeoyl-conjugate (21.77, 271) | 8.1 | 0.7 | 0.0 | 0.0 | 0.00 | 0.000 |
| caffeoyl-shikimate (20.69; 171) | 171 | 11 | 0.0 | 0.0 | 0.00 | 0.000 |

Additional file 6. Metabolite levels in phloem of control and *UGPase2* overexpression lines.

Mean and standard error of the mean (sem) metabolite concentrations (μg g^-1^ fresh weight in sorbitol equivalent response) of phloem tissue of overexpression *UGPase2* transgenic *Populus deltoides* and nontransgenic control plants. The fold change of the metabolite concentrations (average of 3 independent lines with 3 replicates for each line) of *UGPase2* versus control plants and the *P*-value of the contrast as determined by Student’s *t*-tests are shown.
