# Supplementary material for: Metabolic profiling reveals altered sugar and secondary metabolism in response to UGPase overexpression in Populus
Source: BMC Plant Biol. 2014 Oct 7;14:265. doi: 10.1186/s12870-014-0265-8 (PMC4197241; doi:10.1186/s12870-014-0265-8)
Supplement: Additional file 7: — Unknown caffeoyl-glycosides in P. deltoides stem phloem of control plants but absent in overexpressed UGPase2 plants. [file 12870_2014_265_MOESM7_ESM.doc]

**Additional file 7.** **Unknown caffeoyl-glycosides in *P. deltoides* stem phloem of control plants but absent in overexpressed *UGPase2* plants.** (A) GCMS extracted ion trace of m/z 307, typical of caffeoyl conjugates. (B) Electron impact (EI) fragmentation pattern of trimethylsilyl (TMS)-derivative of unknown at RT 20.69 min key m/z 307 171 396 324. (C) EI fragmentation pattern of TMS-derivative of unknown at RT 21.77 min key m/z 307 271 219 324.
