# Supplementary material for: Metabolic profiling reveals altered sugar and secondary metabolism in response to UGPase overexpression in Populus
Source: BMC Plant Biol. 2014 Oct 7;14:265. doi: 10.1186/s12870-014-0265-8 (PMC4197241; doi:10.1186/s12870-014-0265-8)
Supplement: Additional file 8: — Ratio of glycosides to aglycones of phenolics in phloem of control and UGPase2 overexpression lines. [file 12870_2014_265_MOESM8_ESM.docx]

|  | **Control** | | **UGPase2** | | **Fold change** |  |
| --- | --- | --- | --- | --- | --- | --- |
| Metabolite glycoside/aglycone | mean | sem | mean | sem | UGPase2/ Control | P-value |
| salicin/salicyl alcochol | 3.63 | 0.40 | 18.54 | 1.73 | 5.10 | 0.000 |
| salicylic acid-2-O-glucoside/salicylic acid | 0.0049 | 0.0004 | 0.0096 | 0.0015 | 1.97 | 0.000 |
| 2-methoxyhydroquinone-1-O-glucoside/2-methoxyhydroquinone | 7.09 | 0.47 | 10.83 | 0.84 | 1.53 | 0.000 |
| 2,5-dihdroxybenzoic acid-5-O-glucoside/2,5-dihydroxybenzoic acid | 13.24 | 0.75 | 19.56 | 1.35 | 1.48 | 0.000 |
| unknown glucoside peak 1(15.15; 314, 299/unknown (10.51; 284, 314, 299) | 3.81 | 0.19 | 5.18 | 0.40 | 1.36 | 0.001 |
| unknown glucoside peak 2(15.37; 314, 299/unknown (10.51; 284, 314, 299) | 4.19 | 0.20 | 5.64 | 0.56 | 1.35 | 0.006 |
| 2-methoxyhydroquinone-4-O-glucoside/2-methoxyhydroquinone | 11.01 | 0.58 | 14.66 | 0.96 | 1.33 | 0.001 |
| coniferin/coniferyl alcohol | 0.84 | 0.16 | 1.04 | 0.31 | 1.24 | 0.525 |
| 2,3-dihdroxybenzoic acid-3-O-glucoside/2,3-dihydroxybenzoic acid | 9.20 | 0.45 | 10.62 | 0.98 | 1.15 | 0.145 |
| syringin/sinapyl alcohol | 3.28 | 0.26 | 3.15 | 0.37 | 0.96 | 0.761 |
| salicortin/6-hydroxy-2-cyclohexenone-1-carboxylic acid | 28.37 | 1.66 | 26.05 | 1.31 | 0.92 | 0.346 |

Additional file 8. Ratio of glycosides to aglycones of phenolics in phloem of control and *UGPase2* overexpression lines. Mean and standard error of the mean (sem) of metabolite ratio, the fold change of the metabolite concentrations (average of 3 independent lines with 3 replicates for each line) of *UGPase2* versus control plants and the *P*-value of the contrast as determined by Student’s *t*-tests are shown.
