# Supplementary material for: Metabolic profiling reveals altered sugar and secondary metabolism in response to UGPase overexpression in Populus
Source: BMC Plant Biol. 2014 Oct 7;14:265. doi: 10.1186/s12870-014-0265-8 (PMC4197241; doi:10.1186/s12870-014-0265-8)
Supplement: Additional file 9: — Metabolite levels in mature leaf of control and UGPase2 overexpression lines. [file 12870_2014_265_MOESM9_ESM.doc]

| Leaf | Control | | UGPase | | Fold change |  |
| --- | --- | --- | --- | --- | --- | --- |
| Metabolite | Mean | sem | Mean | sem | UGPase/ Control | P-value |
| caffeoyl-glycoside (19.14, 331 171) | 0.0 | 0.0 | 12.0 | 0.8 | infinity | 0.000 |
| caffeoyl-glycoside (19.31; 331 171) | 0.0 | 0.0 | 9.9 | 0.8 | infinity | 0.000 |
| caffeoyl-conjugate (18.14; 171, 331, 463) | 0.7 | 0.3 | 10.5 | 1.8 | 14.94 | 0.000 |
| phenolic (17.5; 171, 219, 331) | 1.2 | 0.4 | 7.8 | 1.6 | 6.23 | 0.000 |
| caffeoyl-glycoside (21.52; 271, 469, 219) | 1.9 | 0.4 | 9.0 | 2.2 | 4.78 | 0.001 |
| 3-O-caffeoylquinic acid | 32 | 6 | 147 | 48 | 4.58 | 0.010 |
| caffeoyl-shikimate (18.24; 171, 219, 463) | 2.3 | 0.4 | 10.1 | 2.7 | 4.34 | 0.002 |
| caffeoyl-shikimate (20.92; 463, 219) | 5.5 | 0.6 | 20.5 | 5.2 | 3.75 | 0.002 |
| cis-3-O-caffeoylquinic acid | 2.7 | 0.3 | 8.8 | 2.0 | 3.23 | 0.001 |
| caffeoyl-shikimate (19.53; 169) | 9.0 | 1.3 | 24.2 | 3.7 | 2.68 | 0.000 |
| citric acid | 707 | 51 | 1872 | 150 | 2.65 | 0.000 |
| caffeoyl-conjugate (19.89; 463, 219, 255) | 2.9 | 0.4 | 7.6 | 1.5 | 2.61 | 0.002 |
| ethyl-phosphate | 267 | 12 | 506 | 35 | 1.90 | 0.000 |
| catechol | 60 | 3 | 92 | 8 | 1.53 | 0.000 |
| glycoside (13.91; 171, 289) | 11 | 1 | 16 | 3 | 1.52 | 0.060 |
| phenolic (23.7; 193, 271, 267, 355, 481, 571) | 212 | 30 | 318 | 73 | 1.50 | 0.153 |
| dihydroxybenzoic acid-galloyl-glycoside (15.83) | 5.9 | 0.5 | 8.4 | 1.0 | 1.43 | 0.020 |
| 2,3-dihydroxybenzoic acid-3-O-glucoside | 6.9 | 0.7 | 9.9 | 1.1 | 1.42 | 0.021 |
| salicyl alcohol | 69 | 4 | 96 | 6 | 1.40 | 0.000 |
| phenolic glycoside (17.23; 179) | 58 | 10 | 79 | 17 | 1.37 | 0.259 |
| salicyloyl-salicortin | 301 | 20 | 399 | 45 | 1.32 | 0.038 |
| phenolic (23.29; 255, 193, 271, 355, 481, 571) | 148 | 21 | 195 | 52 | 1.32 | 0.365 |
| phenolic (23.53; 255, 193, 271, 481, 571) | 113 | 16 | 147 | 42 | 1.30 | 0.404 |
| salicylic acid | 302 | 23 | 367 | 31 | 1.21 | 0.098 |
| phenolic (23.17; 255, 193, 271, 481, 571) | 388 | 61 | 460 | 155 | 1.19 | 0.633 |
| 2,5-dihydroxybenzoic acid-5-O-glucoside | 39 | 3 | 45 | 7 | 1.16 | 0.398 |
| 6-hydroxy-2-cyclohexenone (enol) | 13 | 1 | 13 | 1 | 1.06 | 0.657 |
| sucrose | 9253 | 375 | 9592 | 567 | 1.04 | 0.607 |
| salicin | 1053 | 94 | 1084 | 132 | 1.03 | 0.844 |
| caffeoyl-conjugate (15.35) | 0.5 | 0.2 | 0.5 | 0.2 | 0.99 | 0.988 |
| 6-hydroxy-2-cyclohexenone-1-carboxylic acid | 25 | 2 | 24 | 3 | 0.98 | 0.895 |
| 6-hydroxy-2-cyclohexenone alcohol | 95 | 12 | 92 | 11 | 0.97 | 0.876 |
| α-salicyloylsalicin | 340 | 40 | 321 | 64 | 0.94 | 0.793 |
| caffeic acid | 64 | 8 | 59 | 10 | 0.92 | 0.692 |
| salicortin | 890 | 104 | 744 | 167 | 0.84 | 0.442 |
| quinic acid | 2218 | 252 | 1727 | 413 | 0.78 | 0.294 |
| caffeoyl-conjugate (18.62; 171, 219) | 5.4 | 0.3 | 4.2 | 0.6 | 0.78 | 0.061 |
| salicylic acid-2-O-glucoside | 5.5 | 0.5 | 4.1 | 0.4 | 0.76 | 0.73 |
| glucose | 5402 | 221 | 4116 | 234 | 0.76 | 0.000 |
| coniferin | 1.7 | 0.2 | 1.2 | 0.1 | 0.72 | 0.037 |
| caffeoylpopuloside | 53 | 7 | 31 | 7 | 0.58 | 0.044 |
| syringin | 4.0 | 0.7 | 2.2 | 0.5 | 0.56 | 0.069 |
| glyceric acid | 1594 | 83 | 821 | 164 | 0.51 | 0.000 |
| galactose | 848 | 52 | 410 | 40 | 0.48 | 0.000 |
| 11.16; 218, 335 | 267 | 21 | 127 | 13 | 0.48 | 0.000 |
| caffeoyl-conjugate (19.57; 171, 97, 208) | 10.5 | 0.8 | 5.0 | 0.3 | 0.47 | 0.000 |
| monogalactosylglycerol | 1149 | 152 | 514 | 150 | 0.45 | 0.006 |
| fructose | 4908 | 202 | 1843 | 281 | 0.38 | 0.000 |
| digalactosylglycerol | 985 | 67 | 361 | 39 | 0.37 | 0.000 |
| raffinose | 147 | 14 | 47 | 9 | 0.32 | 0.000 |
| caffeoyl-conjugate (20.31; 171, 219, 331) | 54 | 7 | 16 | 5 | 0.30 | 0.000 |
| shikimic acid | 4004 | 353 | 927 | 292 | 0.23 | 0.000 |
| phenolic glycoside (14.58; 284, 269) | 120 | 8 | 23 | 2 | 0.19 | 0.000 |
| caffeoyl-shikimate (20.69; 171) | 47 | 6 | 5 | 5 | 0.11 | 0.000 |

Additional file 9. Metabolite levels in mature leaf of control and *UGPase2* overexpression lines. Mean and standard error of the mean (sem) metabolite concentrations (μg g^-1^ fresh weight in sorbitol equivalent response) of mature leaf of overexpression *UGPase2* transgenic *Populus deltoides* and nontransgenic control plants. The fold change of the metabolite concentrations (average of 3 independent lines with 3 replicates for each line) of *UGPase2* versus control plants and the *P*-value of the contrast as determined by Student’s *t*-tests are shown.
