# Supplementary material for: Metabolic profiling reveals altered sugar and secondary metabolism in response to UGPase overexpression in Populus
Source: BMC Plant Biol. 2014 Oct 7;14:265. doi: 10.1186/s12870-014-0265-8 (PMC4197241; doi:10.1186/s12870-014-0265-8)
Supplement: Additional file 10: — Unknown caffeoyl-glycosides in P. deltoides leaves unique to overexpressed UGPase2 transgenic lines. [file 12870_2014_265_MOESM10_ESM.doc]

**Additional file 10.** **Unknown caffeoyl-glycosides in *P. deltoides* leaves unique to overexpressed *UGPase2* transgenic lines**. (A) GCMS extracted ion trace of m/z 307, typical of caffeoyl conjugates. (B) EI fragmentation pattern of trimethylsilyl TMS-derivative of unknown at RT 19.14 min key m/z 307 331 171 324. (C) EI fragmentation pattern of TMS-derivative of unknown at RT 19.31 min key m/z 307 331 171 324.
