# Supplementary material for: Metabolic profiling reveals altered sugar and secondary metabolism in response to UGPase overexpression in Populus
Source: BMC Plant Biol. 2014 Oct 7;14:265. doi: 10.1186/s12870-014-0265-8 (PMC4197241; doi:10.1186/s12870-014-0265-8)
Supplement: Additional file 11: — List of gene primer sequences and gene models used in this study. [file 12870_2014_265_MOESM11_ESM.doc]

**Additional file 11. List of gene primer sequences and gene models used in this study.**
